# Serological Investigation of Food Specific Immunoglobulin G Antibodies in Patients with Inflammatory Bowel Diseases

**DOI:** 10.1371/journal.pone.0112154

**Published:** 2014-11-13

**Authors:** Chenwen Cai, Jun Shen, Di Zhao, Yuqi Qiao, Antao Xu, Shuang Jin, Zhihua Ran, Qing Zheng

**Affiliations:** Key Laboratory of Gastroenterology & Hepatology, Ministry of Health, Division of Gastroenterology and Hepatology, Ren Ji Hospital, School of Medicine, Shanghai Jiao Tong University, Shanghai Institute of Digestive Diseases, 145 Middle Shandong Road, Shanghai 200001, China; University of Chicago, United States of America

## Abstract

**Objective:**

Dietary factors have been indicated to influence the pathogenesis and nature course of inflammatory bowel diseases (IBD) with their wide variances. The aim of the study was to assess the prevalence and clinical significance of 14 serum food specific immunoglobulin G (sIgG) antibodies in patients with IBD.

**Methods:**

This retrospective study comprised a total of 112 patients with IBD, including 79 with Crohn's disease (CD) and 33 with ulcerative colitis (UC). Medical records, clinical data and laboratory results were collected for analysis. Serum IgG antibodies against 14 unique food allergens were detected by semi-quantitative enzyme linked immunosorbent assay (ELISA).

**Results:**

Food sIgG antibodies were detected in 75.9% (60/79) of CD patients, 63.6% (21/33) of UC patients and 33.1% (88/266) of healthy controls (HC). IBD patients showed the significantly higher antibodies prevalence than healthy controls (CD *vs.* HC, *P* = 0.000; UC *vs.* HC, *P* = 0.001). However no marked difference was observed between CD and UC groups (*P* = 0.184). More subjects were found with sensitivity to multiple antigens (≥3) in IBD than in HC group (33.9% vs.0.8%, *P* = 0.000). Egg was the most prevalent food allergen. There was a remarkable difference in the levels of general serum IgM (*P* = 0.045) and IgG (*P* = 0.041) between patients with positive and negative sIgG antibodies. Patients with multiple positive allergens (≥3) were especially found with significant higher total IgG levels compared with sIgG-negative patients (*P* = 0.003). Age was suggested as a protective factor against the occurrence of sIgG antibodies (*P* = 0.002).

**Conclusions:**

The study demonstrates a high prevalence of serum IgG antibodies to specific food allergens in patients with IBD. sIgG antibodies may potentially indicate disease status in clinical and be utilized to guide diets for patients.

## Introduction

Inflammatory bowel diseases (IBD) include two main types, Crohn's disease (CD) and ulcerative colitis (UC), both characterized by mucosal ulceration in gut. Patients with IBD suffer from abdominal pain, diarrhea, weight loss and fatigue. As diseases progress, they can cause perianal lesion, abdominal abscess, intestinal perforation or even canceration as diverse complications. Extraintestinal manifestations including oral ulcer, arthritis, iritis and cholangitis may also occur simultaneously [1]. The tendentiousness of the constant alternation of relapses and remissions seriously affects the quality of life in IBD patients [2]. For the moment the mechanisms involved in the pathogenesis can be summarized that the influence of environmental factors to genetically susceptible individuals triggers abnormal mucosal immune reaction in gut with the involvement of intestinal flora and eventually leads to the bowel inflammation and ulceration [3].

IBD has long been regarded as a problem threatening public health in the Western world. However in recent decades the rising incidence of it in developing nations has made IBD a global issue. In Asia, according to an epidemiological survey, the rapid westernization and modernization may attribute to the condition [4]. Among various environmental factors, diet has been implicated to play a considerable role in the course of IBD [5]–[8]. A Japanese study suggested the intake of a high fat diet and sweets may associate with CD and UC [9]. Another research also pointed an increased consumption of alcohol and red meat might cause higher incidence of relapse in UC patients [10]. Meanwhile food intolerance, which is defined as a reproducible adverse reaction to specific food or food ingredients, has become more common in recent years [11]–[12]. Banai J. considered all kinds of food could be antigenic properties to cause chronic mild inflammation in gut and eventually lead to ulcerative colitis [13]. For example, intolerance to milk has long been believed involving in the pathogenesis of IBD [14], [15]. Glassman *et al* reported during infancy stage the frequency of symptoms compatible with milk protein sensitivity was greater in UC compared with control population (*P*<0.03) [16]. Furthermore patients who underwent milk intolerance were found to develop UC at an earlier age compared to those without a history of hypersensitivity to milk (*P*<0.02) [16]. The statistics suggested the early antigenic stimuli might play a role in development of IBD at a later age [17]. However the causality of food intolerance and IBD still remains controversial and needs further researches to figure out.

Studies on food adverse reactions mediated by immunoglobulin G (IgG) in certain intestinal diseases, such as irritable bowel syndrome (IBS), have been increasingly reported [18]–[19]. Researchers used to mainly focus on food intolerance classically by the presence of serous IgE antibodies but it seemed that the characteristically immediate allergic reactions were quite rare in some conditions [20]–[21]. By contrast, the circulating IgG antibodies provide a more delayed or even asymptomatic response after the exposure to a unique food antigen [22]. Considering that IBD patients mostly suffer a long course of the chronic disease, we hypothesize IgG may have a stronger relevance with IBD than IgE.

Food is a complicated field to study because of its enormous varieties. In another way this feature also makes it a resource palace for us researchers to explore. In this study we aimed to identify the prevalence and significance of 14 food specific IgG antibodies in IBD patients through serological investigation. We expected the results may provide a more clear and detailed connection between food intolerance and IBD in our patient population as well as a supporting evidence for the antibody test to serve the clinical.

## Subjects and Methods

### Ethics Statement

This study was approved by Medical Ethics Committee of Ren Ji Hospital of Shanghai Jiao Tong University School of Medicine. Informed consent wasn't applied as the medical records and private information of all the subjects were anonymized and de-identified prior to analysis.

### Subjects

The study included a total of 112 patients with CD (n = 79) or UC (n = 33) in Ren Ji Hospital from June 2011 to December 2013. All patients met the diagnostic criteria for CD or UC according to the consensus on the diagnosis of IBD drawn up by European Crohn's and Colitis Organization (ECCO) [23], [24] and were serologically tested of the food sIgG antibodies during their visit in hospital. In addition another 266 people, who came to our Health Care Centre to do checkups which contained the test as a routine item, were randomly chosen to represent the general population as healthy controls (HC).

### Data Collection

Medical records and clinical data of all the IBD patients were comprehensively reviewed and the general demographic data were summarized. The disease severity of CD or UC was based on Harvey-Bradshaw Index [25] or Modified Truelove-Witts Classification [26], respectively. The disease localization of both CD and UC was determined by Montreal Classification [27]. The complications that patients carried consisted of fistula, abdominal abscess, intestinal obstruction, perianal disease, hemorrhage of gastrointestinal tract and acute perforation. The simultaneous extraintestinal manifestations included oral ulcer, sacro-iliitis, rheumatoid arthritis, iritis, primary sclerotic cholangitis, hepatic adipose infiltration and cholelithiasis. Relevant laboratory findings contained peripheral white blood cell (WBC) counts, eosinophile granulocyte (EOS) counts, lymphocyte (LYM) counts, haemoglobin (Hb), serum albumin, erythrocyte sedimentation rate (ESR), high-sensitivity C-reactive protein (hs-CRP), serum total immunoglobulin (IgM, IgA and IgG) and anti double-stranded DNA antibodies (anti-dsDNA).

### Enzyme Linked Immunosorbent Assay (ELISA)

ELISA tests for the semi-quantitative analysis of serum IgG antibodies to 14 unique food allergens, including rice, egg, mushroom, milk, pork, chicken, beef, crab, codfish, corn, soybean, tomato, shrimp and wheat, were performed by the detection kit according to the operation manual (Biomerica, Inc. USA). The IgG concentration less than 50U/ml was considered negative (Grade 0). The values between 50–100U/ml, 100–200U/ml and more than 200U/ml were represented mild sensitivity (Grade +1), moderate sensitivity (Grade +2) and high sensitivity (Grade +3), respectively.

### Data Analysis

Statistics were performed with SPSS 19.0 (SPSS Inc. USA). Enumeration data were analyzed using chi-squared test, in which rates of multiple samples were compared by R×C contingency table analysis. Continuous numerical variables were expressed in the form of mean with 95% confidence interval (95% CI) and analyzed by Student's *t* test. Regression analysis was utilized to identify the correlation/risk factors among variables. Two-tailed *P*-value<0.05 was regarded statistically significant.

## Results

### Demographic data of all subjects

The characteristic information of IBD patients and healthy controls were summarized in **Table 1**.

**Table 1:** Demographic data of all subjects.

Clinicopathological features	CD (N = 79)	UC (N = 33)	HC (N = 266)
Male (*n*, %)	47(59.5)	18(54.5)	146(54.9)
Female (*n*, %)	32(40.5)	15(45.5)	120(45.1)
Age (yr) (mean, 95% CI)	36.5(33.6–39.4)	40.7 (35.9–45.7)	46.4(45.2–47.6)
Age range (yr)	18–68	17–73	24–71
Duration of disease (yr) (*n*, %)			
<1	21 (26.6)	13 (39.4)	/
1–5	38 (48.1)	11 (33.3)	/
5–10	16 (20.3)	7 (21.2)	/
>10	4 (5.1)	2 (6.1)	/
Disease activity (*n*, %)			
Remission	7 (8.9)	0 (0)	/
Mild	16 (20.3)	12 (36.4)	/
Moderate	33 (41.8)	14 (42.4)	/
Severe	23 (29.1)	7 (21.2)	/
Localization of disease (*n*, %)			
L1 (terminal ileum)	40 (50.6)	/	/
L2 (colon)	10 (12.7)	/	/
L3 (ileocolon)	29 (36.7)	/	/
E1 (rectum)	/	1 (3.0)	/
E2 (left-sided colon)	/	14 (42.4)	/
E3 (entire colon)	/	18 (54.5)	/
Complications of disease (*n*, %)			
None	32 (40.5)	31 (93.9)	/
1 item	42 (53.2)	2 (6.1)	/
2 items	5 (6.3)	/	/
Extraintestinal manifestations (*n*, %)			
None	60 (75.9)	26 (78.8)	/
1 item	18 (22.8)	7 (21.2))	/
2 items	1 (1.3)	/	/
Intestinal surgery	15 (19.0)	0 (0.0)	/

CD: Crohn's disease; UC: ulcerative colitis; HC: healthy controls; yr: year; 95% CI: 95% confidence interval; L1,L2,L3: disease localization of Crohn's disease by Montreal Classification; E1,E2,E3: disease localization of ulcerative colitis by Montreal Classification.

### Prevalence of serum IgG antibodies to 14 unique food allergens

**Figure 1:**
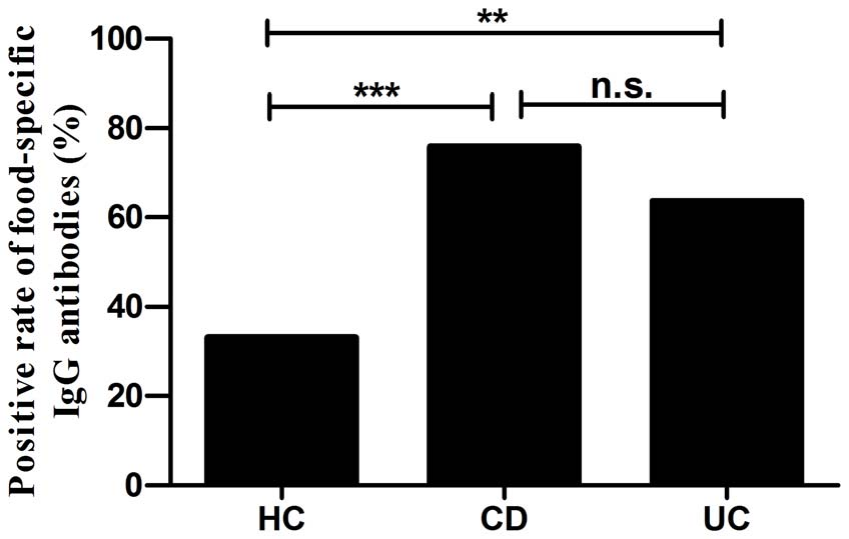
Positive rate of food-specific IgG antibodies in Crohn's disease (CD), ulcerative colitis (UC) and healthy control (HC) groups. Chi-square test, *** *P*<0.001, **P<0.005, n.s. not significant.

**Figure 2:**
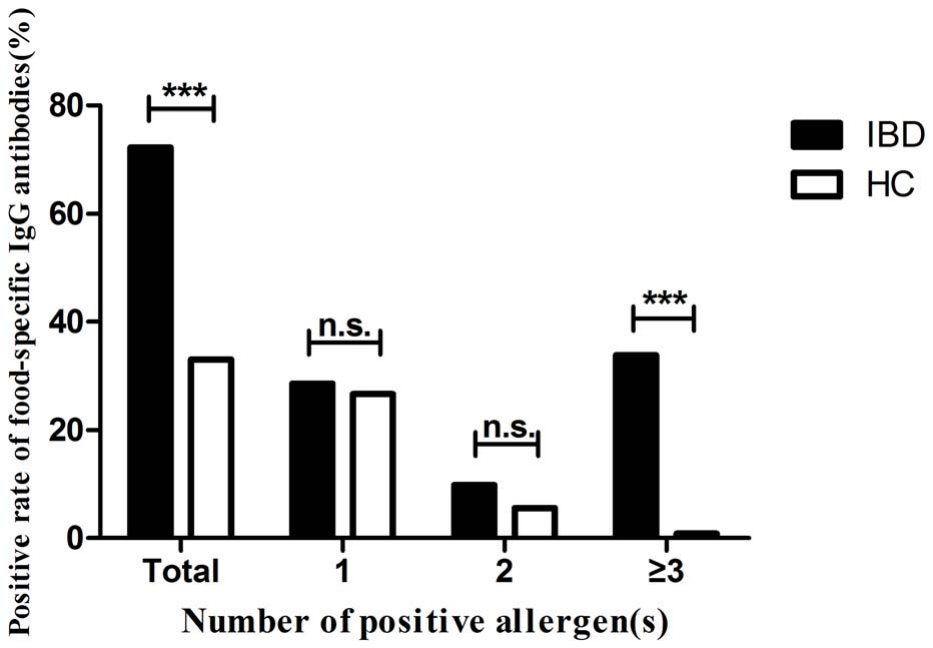
Distribution of the number of positive allergen(s) with positive rate of food-specific IgG antibodies in patients with inflammatory bowel diseases (IBD) and healthy controls (HC). Chi-square test, *** *P*<0.001, n.s. not significant.

Food specific IgG antibodies were detected positive in 75.9% (60/79) of CD patients, 63.6% (21/33) of UC patients and 33.1% (88/266) of HC (**Table 2**). The antibodies showed a significantly higher frequency in both CD and UC groups than in healthy controls (CD *vs* HC, *P* = 0.000; UC *vs* HC, *P* = 0.001) (**Figure 1**). However, there was no significant difference between CD and UC groups (*P* = 0.184). In general the total positive rate of all IBD patients was 72.3% (81/112), higher than the control group (*P* = 0.000). Among them 28.6% (32/112), 9.8% (11/112) and 33.9% (38/112) of subjects were respectively sensitive to one, two and more than two food allergens while the corresponding ratios of healthy controls were 26.7% (71/266), 5.6% (15/266) and 0.8% (2/266). There were more subjects who got intolerant to 3 or more antigens in IBD group than in HC group (*P* = 0.000) (**Figure 2**).

**Table 2:** Prevalence of food specific IgG antibodies in patients with Crohn's disease (CD), ulcerative colitis (UC) and healthy controls (HC).

Group	*N*	Seropositive Degree (*n*)				Antibodies (−)	Antibodies (+)
		0	+1	+2	+3	(*n*, %)	(*n*, %)
CD	79	19	10	14	36	19 (24.1)	60 (75.9)
UC	33	12	9	4	8	12 (36.4)	21 (63.6)
HC	266	178	41	24	23	178 (66.9)	88 (33.1)

**Figure 3:**
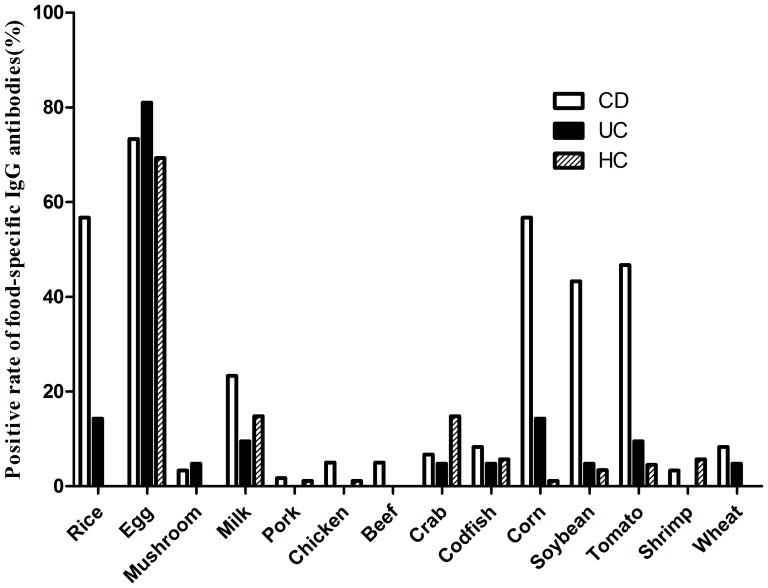
Distribution of positive food allergens in Crohn's disease (CD), ulcerative colitis (UC) and healthy control (HC) groups.

In the present study, the top five prevalent food allergens which caused positive sIgG antibodies in CD patients were egg (44/60, 73.3%), rice (34/60, 56.7%), corn (34/60, 56.7%), tomato (28/60, 46.7%) and soybean (26/60, 43.3%). Besides, the top five prevalent food allergens in UC group were egg (17/21, 81.0%), rice (3/21, 14.3%), corn (3/21, 14.3%), tomato (2/21, 9.5%) and milk (2/21, 9.5%). And healthy controls demonstrated the compositions of egg (61/88, 69.3%), milk (13/88, 14.8%), crab (13/88, 14.8%), codfish (5/88, 5.7%) and shrimp (5/88, 5.7%), which were similar to the results of a reported epidemiological survey in general population [28]. **Figure 3** showed the distribution of positive food allergens in CD, UC and HC groups.

### Association of food specific IgG antibodies with inflammatory segments

To analyze the association between food sIgG antibodies status and disease extent in gut, all 112 patients were divided into three subgroups according to endoscopic results (**Table 3**). The positive rate was found higher (82.5%) in patients with only small intestine involved but the statistical difference wasn't remarkable (*P* = 0.072).

**Table 3:** Distribution of food specific IgG antibodies in different disease localizations.

Localization	sIgG antibodies (+)	sIgG antibodies (−)
	(*n*, %)	(*n*, %)
Only small intestine (*N* = 40)	33 (82.5)	3 (17.5)
Only large intestine (*N* = 43)	26 (60.5)	17 (39.5)
Both small & large intestine (*N* = 29)	22 (75.9)	7 (24.1)
*P*-value	0.072^▵^	

▵ Chi-square test (R×C contingency table analysis), not statistically significant (*P*>0.05).

### Relevance of food specific IgG antibodies with disease activity

We divided all 112 IBD patients into four subgroups according to clinical disease activity and compared the ratios of patients with positive IgG antibodies, multiple positive antibodies (≥2) and high sensitivity to at least one food allergen (**Table 4**). Although the general antibodies and multi-antibodies were found more common in remission group (85.7% and 71.4%) while the patients who were highly sensitive to specific food allergens seemed to be severer (46.7%), there was no significant differences among the four subgroups.

**Table 4:** Distribution of food specific IgG antibodies at different IBD activity status.

Disease status	sIgG antibodies (+)	Multiple positive (≥2)	High sensitivity
	*n* (%)	*n* (%)	*n* (%)
Remission (*N* = 7)	6 (85.7)	5 (71.4)	3 (42.9)
Mild (*N* = 28)	18 (64.3)	7 (25)	9 (32.1)
Moderate (*N* = 47)	35 (74.5)	23 (48.9)	18 (38.3)
Severe (*N* = 30)	22 (73.3)	14 (46.7)	14 (46.7)
*P*-value	0.647▵	0.079▵	0.719▵

▵ Chi-square test (R×C contingency table analysis), not statistically significant (*P*>0.05).

To further analyze the possible association with several laboratory values related to disease activity, IBD patients were classified into positive and negative IgG antibodies subgroups (**Table 5**). We did find a notable difference in general serum IgM (*P* = 0.045) and IgG (*P* = 0.041) levels between the two groups. Mean values of WBC, ESR and hs-CRP in the positive patients were recognized higher compared with the other group although no marked differences were found. The rest lab findings of the two groups were resembled.

**Table 5:** Comparison of laboratory results in IBD patients with positive and negative food specific IgG antibodies.

Laboratory results	sIgG antibodies (+)	sIgG antibodies (−)	*P*-value
	(N = 81)	(N = 31)	
WBC (×10^9^/L)	7.15(6.50–7.86)	6.66(5.79–7.56)	0.437
EOS (×10^9^/L)	0.16(0.12–0.23)	0.15(0.10–0.21)	0.833
LYM (×10^9^/L)	1.44(1.31–1.59)	1.54(1.32–1.81)	0.501
Hb (g/L)	114.31(109.52–119.06)	114.23(104.84–122.58)	0.986
Albumin (g/L)	36.19(34.82–37.72)	35.98(33.67–38.28)	0.889
ESR (mm/h)	28.07(23.01–33.58)	21.35(15.65–27.61)	0.160
hs-CRP (mg/L)	18.84(13.78–23.89)	11.75(6.51–18.51)	0.083
IgM (g/L)	1.22(0.99–1.49)	0.79(0.60–1.03)	0.045*
IgA (g/L)	2.80(2.37–3.27)	2.60(2.13–3.05)	0.597
IgG (g/L)	12.90(11.94–13.94)	10.92(9.50–12.48)	0.041*
anti-dsDNA (IU/mL)	3.31(2.85–3.75)	3.56(2.72–4.61)	0.609

Statistics were expressed as mean with 95% confidence interval.

Normal range: white blood cell (WBC), 3.69–9.16×10^9^/L; eosinophile granulocyte (EOS), 0.02–0.50×10^9^/L; lymphocyte (LYM), 0.8–4.0×10^9^/L; haemoglobin (Hb), 113–172 g/L; albumin, 35–55 g/L; erythrocyte sedimentation rate (ESR) 0–20 mm/h; high-sensitivity C-reactive protein (hs-CRP), 0–3 mg/L; immunoglobulin M (IgM) 0.4–2.3 g/L; IgA, 0.7–4.0 g/L; IgG, 7–16 g/L; anti double-stranded DNA antibodies (anti-dsDNA), 0–7.0 IU/mL

Student's *t* test, *P<0.05.

**Figure 4:**
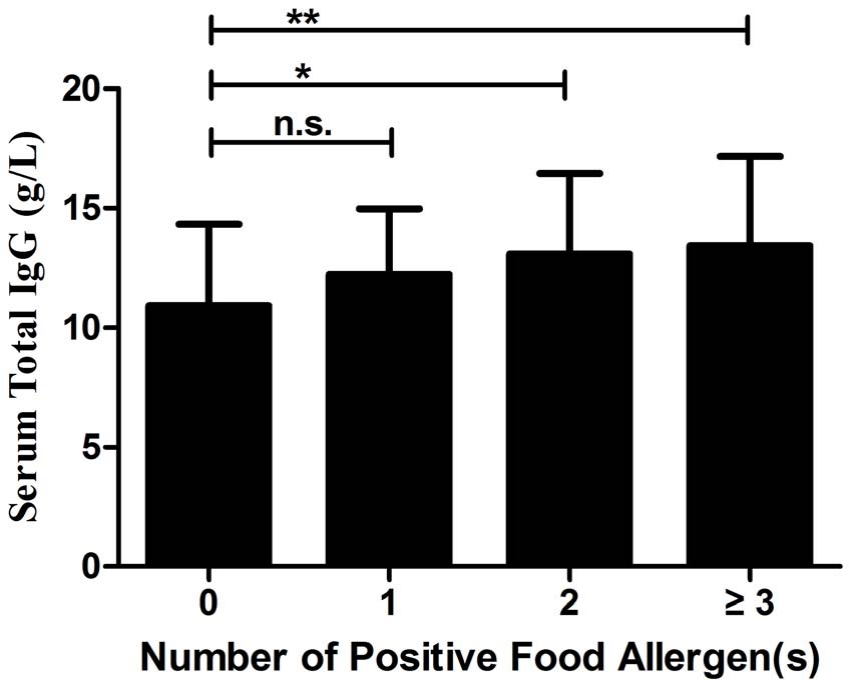
Serum total IgG values in inflammatory bowel disease patients with multiple positive food allergens. Statistics were shown as mean ± standard deviation. Chi-square test, * *P*<0.05; ** *P*<0.005, n.s. not significant.

Considering the relative levels of total IgG in relation to antigen specific IgG, we then divided all the IBD patients into 4 groups according to the number of positive allergen(s) (0, 1, 2, ≥3) and compared the difference of serum general IgG values among the groups (**Figure 4**). The mean values of each group were 10.92 (9.50–12.48) g/L, 12.24 (11.31–13.32) g/L, 13.08 (11.31–14.97) g/L and 13.42 (12.24–14.58) g/L, respectively. Patients with multiple positive allergens (≥2) were found with significant higher total IgG levels than sIgG-negative patients (*P* = 0.044, *P* = 0.003, respectively).

### Predictors for the occurrence of food specific IgG antibodies

To further identify the predictive factors, we utilized binary logistic regression analyses of several demographic and clinical parameters. The independent variables contained disease type (CD/UC), gender, age, complication, extraintestinal manifestation and intestinal surgery (**Table 6**). Only age was found as a predisposing as well as protective factor against the development of serum food-related IgG antibodies (*P* = 0.002).

**Table 6:** Correlation of food specific IgG antibodies with demographic and clinical parameters.

Parameters	Odds ratio	95% CI	*P*-value
Disease type (CD vs. UC)	1.167	0.389–3.498	0.783
Gender	0.758	0.306–1.879	0.549
Age	0.945	0.912–0.979	0.002**
Complication	2.269	0.719–7.157	0.162
Extraintestinal manifestation	1.302	0.438–3.868	0.635
Intestinal surgery	1.613	0.329–7.905	0.556

95% CI: 95% confidence interval; CD: Crohn's disease; UC: ulcerative colitis.

Binary logistic regression, **P<0.005.

## Discussion

In the present study, we have demonstrated that IgG antibodies against food specific allergens are distinctly elevated in IBD patients compared with healthy controls. This serologic antibody investigation is served as an aid in the diagnosis and management of food intolerance in clinical activities [29]. Food intolerance can be defined as a series of unpleasant symptoms including eructation, abdominal pain, diarrhea, fatigue, headache and palpitation after the intake of particular food products [29]. It is usually caused by enzyme deficiency as well as pharmacological effects of vasoactive amines present in foods. When certain food can't be fully digested, bodies may produce specific IgG antibodies which would form immune complexes with food particles and lead to excessive protective immune responses [30]. Food intolerance is not so similar to food allergy as the latter mainly occurs through the classically immediate IgE-mediated antibody responses and the obvious symptoms can be detected by most patients [31]. Meanwhile, IgG-mediated reaction characteristically acts as a particular delayed-type hypersensitivity response after the exposure to antigens and sometimes the symptoms are too occult for patients to recognize [22]. Some investigators considered them to be physiological since that the food sIgG antibodies can appear in healthy individuals [32], which was also proved in our data. However, several studies including our results have shown the higher prevalence of sIgG antibodies to food in IBD patients or animal models in contrast to normal controls [19], [33], which arouse the attention to the relevance between IBD and food intolerance. Van Den Bogaerde *et al.* used colonoscopic allergen provocation test to determine the gut mucosal response to food antigens in Crohn's disease [34]. This intuitionistic study provided both *in vivo* and *in vitro* evidence that CD patients were more sensitive to exogenous food antigens than healthy people and the reactions were gut specific.

Food sIgG antibodies were discovered frequent in IBD patients with small intestine involved in the present research, which resembled the result of a study on the correlation of lactose malabsorption and disease segments [35]. Mishkin *et al.* demonstrated that CD of the proximal small bowel (duodenum, jejunum), terminal ileum, terminal ileum plus colon and colon alone were related with lactose malabsorption of 100%, 68.1%, 54.5% and 43.5%, respectively. Regression analysis of our data indicated age as a protective factor of food intolerance in IBD patients, which is opposite to the result of a cross sectional epidemiological study in other area of China [28]. We assume the difference might be associated with age structure of onset as inflammatory bowel diseases, especially Crohn's disease, tend to occur in younger people based on our clinical observation.

A survey demonstrated that 15.6% IBD patients believed diet could initiate the disease while 57.8% were convinced certain foods could play a role in causing relapses [36]. In our study, we found food sIgG antibodies were more frequent in patients who were during remission. Meanwhile, the patients who were highly sensitive to food allergens tended to be serious in disease activity and mean levels of ESR and hs-CRP were detected higher in sIgG-positive patients. Although the results didn't show statistical significance, they did provide us with a tendency. We consider the few remission cases (only 7) contributed to the above conflicting results.

The increased serum IgM values tested in sIgG-positive patients may indicate recent infection. Besides, we also found a higher level of serum total IgG antibodies in patients with positive allergens, especially with multiple ones in the study. The general increase on IgG levels may be an important reason for the increased amount of sIgG antibodies against food epitopes. It has been reported that mast cells can respond not only to IgE antibodies but also to IgG antibodies [37]. In addition IgG antibodies in food allergy may even influence allergen-IgE complex formation and bind to B cells, which is quite opposite the traditional concept that IgG antibodies are supposed to inhibit these processes [38]. Therefore IgG antibodies probably serve as mediation effects rather than inhibition in hypersensitivity reaction caused by food intolerance. Serum sIgG antibodies to non-food related antigens are mostly studied within inhaled antigens in respiratory allergic disorders such as asthma. Wang *et al.* investigated the relevance between asthma morbidity and sIgE/sIgG levels to inhaled allergen exposure [39]. They concluded that sIgE levels could serve as markers of asthma however sIgG was not that important as predictor or modifier. Due to the rich diversity of food and non-food allergens, we believe that the immune reactions can be very distinct for different allergens and the mechanisms of how sIgG functions may be complicated and needs further study.

How IBD and food intolerance interact with each other remains controversial. Disruption of epithelial tight junctions causes hyperpermeability in the gut of IBD patients and allows the antigen presenting cells (dendritic cells) to directly encounter food antigens in lamina propria to activate Th/B cells, thus resulting in high levels of sIgG antibodies [40]. Simultaneously this defect may also exacerbate inflammatory conditions. In another respect, lack of certain enzyme may lead to incomplete digestion of food and the remaining polypeptides then stimulate the secretion of sIgG antibodies as well as inflammatory cytokines, thereby bringing impairments to normal intestines [41]. The definite mechanism still remains unclear.

Although we used several methods to improve our study, there were still some limitations to the present results. The small quantity of cases may bring about the first shortcoming. The sample size needs to be enlarged to see the exact trend. We didn't contain the follow-up data of whether or not the IBD patients eliminated the food based on the presence of sIgG antibodies and whether the subsequent diet elimination had an effect on the diseases, which is also a limitation of our research. However, promising results have been reported in a randomized controlled trial [19]. Bentz *et al.* designed a double-blind, cross-over nutritional diet intervention according to circulating food sIgG antibodies with 40 CD patients and it did show therapeutic effects with regard to abdominal pain, stool frequency and general well-being. However, the investigators could not elucidate the exact mechanisms of the contribution of sIgG antibodies to disease activity. Anyway, we still consider serologic investigation of IgG antibodies to food antigens may bring benefits to dietary modification for IBD patients.

In conclusion, the prevalence of food specific IgG antibodies is remarkably higher in patients with inflammatory bowel diseases than healthy controls, and age may acts as a protective factor for their occurrence. Although the mechanisms of the interaction between food intolerance and IBD remain obscure, the sIgG antibodies may potentially show the clinical significance to indicate disease status and to ameliorate symptoms by guiding diets for patients.

## References

[1] Bernstein CN (2001) Extraintestinal manifestations of inflammatory bowel disease. Curr Gastroenterol Rep 3: 477–483. 11696285 10.1007/s11894-001-0068-6

[2] Vidal A, Gomez-Gil E, Sans M, Portella MJ, Salamero M, et al (2008) Health-related quality of life in inflammatory bowel disease patients: the role of psychopathology and personality. Inflamm Bowel Dis 14: 977–983. 18275078 10.1002/ibd.20388

[3] Macdonald TT, Monteleone G (2005) Immunity, inflammation, and allergy in the gut. Science 307: 1920–1925. 15790845 10.1126/science.1106442

[4] Goh K, Xiao SD (2009) Inflammatory bowel disease: a survey of the epidemiology in Asia. J Dig Dis 10: 1–6. 19236540 10.1111/j.1751-2980.2008.00355.x

[5] Magee EA, Edmond LM, Tasker SM, Kong SC, Curno R, et al (2005) Associations between diet and disease activity in ulcerative colitis patients using a novel method of data analysis. Nutr J 4: 7. 15705205 10.1186/1475-2891-4-7 PMC549081

[6] Hou JK, Abraham B, El-Serag H (2011) Dietary intake and risk of developing inflammatory bowel disease: a systematic review of the literature. Am J Gastroenterol 106: 563–573. 21468064 10.1038/ajg.2011.44

[7] Brunner B, Scheurer U, Seibold F (2007) Differences in yeast intolerance between patients with Crohn's disease and ulcerative colitis. Dis Colon Rectum 50: 83–88. 17096175 10.1007/s10350-006-0749-1

[8] Haboubi NY, Jones S (2005) Influence of dietary factors on the clinical course of inflammatory bowel disease. Gut 54: 567. PMC1774464 15753551

[9] Sakamoto N, Kono S, Wakai K, Fukuda Y, Satomi M, et al (2005) Dietary risk factors for inflammatory bowel disease: a multicenter case-control study in Japan. Inflamm Bowel Dis 11: 154–163. 15677909 10.1097/00054725-200502000-00009

[10] Jowett SL, Seal CJ, Pearce MS, Phillips E, Gregory W, et al (2004) Influence of dietary factors on the clinical course of ulcerative colitis: a prospective cohort study. Gut 53: 1479–1484. 15361498 10.1136/gut.2003.024828 PMC1774231

[11] Sampson HA (2004) Update on food allergy. J Allergy Clin Immunol 113: : 805–819; quiz 820. 10.1016/j.jaci.2004.03.014 15131561

[12] David TJ (2000) Adverse reactions and intolerance to foods. Br Med Bull 56: 34–50. 10885103 10.1258/0007142001902950

[13] Banai J (2009) Nutrition in inflammatory bowel disease. Orv Hetil 150: 839–845. 19383575 10.1556/OH.2009.28599

[14] Binder JH, Gryboski JD, Thayer WR Jr, Spiro HM (1966) Intolerance to milk in ulcerative colitis. A preliminary report. Am J Dig Dis 11: 858–864. 6012544 10.1007/BF02233942

[15] Truelove SC (1961) Ulcerative colitis provoked by milk. Br Med J 1: 154–160. 13778258 10.1136/bmj.1.5220.154 PMC1952962

[16] Glassman MS, Newman LJ, Berezin S, Gryboski JD (1990) Cow's milk protein sensitivity during infancy in patients with inflammatory bowel disease. Am J Gastroenterol 85: 838–840. 2371984

[17] Cashman KD, Shanahan F (2003) Is nutrition an aetiological factor for inflammatory bowel disease? Eur J Gastroenterol Hepatol 15: 607–613. 12840670 10.1097/00042737-200306000-00005

[18] Atkinson W, Sheldon TA, Shaath N, Whorwell PJ (2004) Food elimination based on IgG antibodies in irritable bowel syndrome: a randomised controlled trial. Gut 53: 1459–1464. 15361495 10.1136/gut.2003.037697 PMC1774223

[19] Bentz S, Hausmann M, Piberger H, Kellermeier S, Paul S, et al (2010) Clinical relevance of IgG antibodies against food antigens in Crohn's disease: a double-blind cross-over diet intervention study. Digestion 81: 252–264. 20130407 10.1159/000264649

[20] Zar S, Kumar D, Benson MJ (2001) Food hypersensitivity and irritable bowel syndrome. Aliment Pharmacol Ther 15: 439–449. 11284772 10.1046/j.1365-2036.2001.00951.x

[21] Mekkel G, Barta Z, Ress Z, Gyimesi E, Sipka S, et al (2005) Increased IgE-type antibody response to food allergens in irritable bowel syndrome and inflammatory bowel diseases. Orv Hetil 146: 797–802. 17918636

[22] Crowe SE, Perdue MH (1992) Gastrointestinal food hypersensitivity: basic mechanisms of pathophysiology. Gastroenterology 103: 1075–1095. 1499910 10.1016/0016-5085(92)90047-3

[23] Stange EF, Travis SP, Vermeire S, Reinisch W, Geboes K, et al (2008) European evidence-based Consensus on the diagnosis and management of ulcerative colitis: Definitions and diagnosis. J Crohns Colitis 2: 1–23. 21172194 10.1016/j.crohns.2007.11.001

[24] Van Assche G, Dignass A, Panes J, Beaugerie L, Karagiannis J, et al (2010) The second European evidence-based Consensus on the diagnosis and management of Crohn's disease: Definitions and diagnosis. J Crohns Colitis 4: 7–27. 21122488 10.1016/j.crohns.2009.12.003

[25] Harvey RF, Bradshaw JM (1980) A simple index of Crohn's-disease activity. Lancet 1: 514. 6102236 10.1016/s0140-6736(80)92767-1

[26] Truelove SC, Witts LJ (1955) Cortisone in ulcerative colitis; final report on a therapeutic trial. Br Med J 2: 1041–1048. 13260656 10.1136/bmj.2.4947.1041 PMC1981500

[27] Satsangi J, Silverberg MS, Vermeire S, Colombel JF (2006) The Montreal classification of inflammatory bowel disease: controversies, consensus, and implications. Gut 55: 749–753. 16698746 10.1136/gut.2005.082909 PMC1856208

[28] Sai XY, Zheng YS, Zhao JM, Zhao W (2011) A cross sectional survey on the prevalence of food intolerance and its determinants in Beijing, China. Chin J Epidemiol 32(3): 302–05 (in Chinese).. 21457670

[29] Palmieri B, Esposito A, Capone S, Fistetto G, Iannitti T (2011) Food intolerance: reliability and characteristics of different diagnostic alternative tests. Minerva Gastroenterol Dietol 57: 1–10. 21785406

[30] Ortolani C, Pastorello EA (2006) Food allergies and food intolerances. Best Pract Res Clin Gastroenterol 20: 467–483. 16782524 10.1016/j.bpg.2005.11.010

[31] Wuthrich B (2009) Food allergy, food intolerance or functional disorder. Praxis (Bern 1994) 98: 375–387. 19340768 10.1024/1661-8157.98.7.375

[32] Husby S, Oxelius VA, Teisner B, Jensenius JC, Svehag SE (1985) Humoral immunity to dietary antigens in healthy adults. Occurrence, isotype and IgG subclass distribution of serum antibodies to protein antigens. Int Arch Allergy Appl Immunol 77: 416–422. 4018884 10.1159/000233819

[33] Foster AP, Knowles TG, Moore AH, Cousins PD, Day MJ, et al (2003) Serum IgE and IgG responses to food antigens in normal and atopic dogs, and dogs with gastrointestinal disease. Vet Immunol Immunopathol 92: 113–124. 12730012 10.1016/s0165-2427(03)00033-3

[34] Van Den Bogaerde J, Cahill J, Emmanuel AV, Vaizey CJ, Talbot IC, et al (2002) Gut mucosal response to food antigens in Crohn's disease. Aliment Pharmacol Ther 16: 1903–1915. 12390099 10.1046/j.1365-2036.2002.01360.x

[35] Mishkin B, Yalovsky M, Mishkin S (1997) Increased prevalence of lactose malabsorption in Crohn's disease patients at low risk for lactose malabsorption based on ethnic origin. Am J Gastroenterol 92: 1148–1153. 9219788

[36] Zallot C, Quilliot D, Chevaux JB, Peyrin-Biroulet C, Gueant-Rodriguez RM, et al (2013) Dietary beliefs and behavior among inflammatory bowel disease patients. Inflamm Bowel Dis 19: 66–72. 22467242 10.1002/ibd.22965

[37] Malbec O, Daeron M (2007) The mast cell IgG receptors and their roles in tissue inflammation. Immunol Rev 217: 206–221. 17498061 10.1111/j.1600-065X.2007.00510.x

[38] Meulenbroek LA, de Jong RJ, den Hartog Jager CF, Monsuur HN, Wouters D, et al (2013) IgG antibodies in food allergy influence allergen-antibody complex formation and binding to B cells: a role for complement receptors. J Immunol 191: 3526–3533. 23997216 10.4049/jimmunol.1202398

[39] Wang J, Visness CM, Calatroni A, Gergen PJ, Mitchell HE, et al (2009) Effect of environmental allergen sensitization on asthma morbidity in inner-city asthmatic children. Clin Exp Allergy 39: 1381–1389. 19489919 10.1111/j.1365-2222.2009.03225.x PMC4785875

[40] Chahine BG, Bahna SL (2010) The role of the gut mucosal immunity in the development of tolerance against allergy to food. Curr Opin Allergy Clin Immunol 10: 220–225. 20431370 10.1097/ACI.0b013e32833982ab

[41] Isolauri E, Rautava S, Kalliomaki M (2004) Food allergy in irritable bowel syndrome: new facts and old fallacies. Gut 53: 1391–1393. 15361481 10.1136/gut.2004.044990 PMC1774228

